# Associations Between Short‐Term Changes in Disease Activity and Long‐Term Clinical Outcomes in Activated Phosphoinositide 3‐Kinase Delta Syndrome

**DOI:** 10.1155/jimr/1486611

**Published:** 2026-09-16

**Authors:** Joanne Tutein Nolthenius, Barbara Torlinska, Louise Linsell, Neil Hawkins, Yuxian Chen, John Whalen, Anna Šedivá

**Affiliations:** ^1^ Pharming Group N.V., Leiden, Netherlands; ^2^ Visible Analytics, Oxford, UK; ^3^ Department of Immunology, University Hospital Motol and Homolka, 2nd Faculty of Medicine, Charles University, Prague, Czech Republic, cuni.cz

**Keywords:** APDS, clinical outcomes, disease activity endpoints, infection rates, PI3Kδ

## Abstract

**Background:**

Clinical trials in activated phosphoinositide 3‐kinase delta (PI3Kδ) syndrome (APDS), an ultra‐rare disease, have evaluated treatment response to the PI3Kδ inhibitor leniolisib using disease activity endpoints, including those measuring immunodeficiency and immune dysregulation. However, the relationship between disease activity endpoints and long‐term clinical outcomes has not been quantified.

**Methods:**

Data from a randomised controlled trial and its open‐label extension (OLE) investigating leniolisib were used to evaluate associations between short‐term disease activity endpoints and longer‐term clinical outcomes. Both single time point and repeated measures analyses were conducted using linear regression modelling and random effects multilevel regression modelling, respectively.

**Results:**

In total, 39 patients were included (≤6 years of follow‐up). Improvements in multiple immunodeficiency and dysregulation disease activity endpoints were significantly associated with increases in the patient global assessment (PtGA) and the 36‐item short form survey (SF‐36) physical component score and mental component score (PCS/MCS). Increasing proportion of naïve B cells was associated with reduction in infection rates (2.3% fewer infections per 1% increase in naïve B cells).

**Conclusions:**

In this clinical trial setting, short‐term improvements in immunodeficiency and dysregulation following treatment with leniolisib were associated with longer‐term improvements in clinical outcomes, including infection rates and quality of life, for patients with APDS.

**Plain Language Summary**

• What is activated phosphoinositide 3‐kinase delta (PI3Kδ) syndrome (APDS)?
  APDS is an ultra‐rare disease in which the immune system does not work correctly. People with APDS can have a wide range of symptoms, which can include infections, an ongoing cough, muscle aches and tiredness, and specific organs of the immune system becoming larger. As these symptoms can start in early childhood, they can lead to a poor quality of life from a young age.
• Why was this study carried out?
  APDS was only recently identified as a disease, so it is not certain how short‐term treatment effects on the immune system might impact people’s health in the long run. To find out more, we looked at data from clinical trials of a treatment for APDS, called leniolisib. In the trials, researchers tested the immune systems of people with APDS after treatment with leniolisib, to see whether the types of cells in their immune system and the size of their immune system organs had changed. Participants also filled out questionnaires about their health over time. By looking at these data together, we hoped to link short‐term treatment effects on the immune system with long‐term health for people with APDS.
• What were the results of this study?
  Researchers were able to find some links between short‐term treatment effects on the immune system and the overall health of participants. For example, when patients had increased numbers of naïve B cells (a type of immune cell), they were likely to have fewer infections in the following years. Other improvements in the cells and size of organs in the immune system were linked to improvements in how well participants felt and their overall physical and mental health.
• What do these results mean?
  These results show that if treatments like leniolisib can improve the immune systems of people with APDS in the short term, it might lead to them having better long‐term health. Researchers could use this information to help develop and test new treatments for APDS, which may help people with APDS to feel better in the long run.

## 1. Introduction

Activated phosphoinositide 3‐kinase delta (PI3Kδ) syndrome (APDS) is an ultra‐rare genetic disorder classified as an inborn error of immunity (IEI) [1, 2]. APDS arises from hyperactive signalling within the PI3Kδ pathway, which is mainly expressed in lymphocytes and is central in regulating lymphocyte development, maturation and proliferation [3–6]. In APDS, hyperactive PI3Kδ prevents B cells from maturing in lymphatic tissue, resulting in elevated numbers and accumulation of transitional B cells and reduced naïve B cells. In addition, hyperactive PI3Kδ prevents class‐switch recombination, resulting in high levels of immunoglobulin M (IgM), with low levels of the high‐affinity Igs required for fighting infections. T cells are also affected, with senescent and exhausted effector T‐cell numbers being elevated in APDS [3, 4, 7–12].

As fundamental cellular processes within the immune system are altered in APDS, a broad range of manifestations accumulate over time [4, 13]. The immune system is unable to mount an effective response to antigens due to impaired T‐cell responses and a lack of high‐affinity antibodies, resulting in severe and recurrent sinopulmonary infections [7, 9, 11, 14, 15]. The imbalance of B and T cells also increases susceptibility to cytomegalovirus (CMV) and Epstein–Barr virus (EBV) infection, while promoting lymphoproliferation (primarily resulting in lymphadenopathy and organomegaly, and also extending to nodular lymphoid hyperplasia affecting the gastrointestinal tract and lungs) as well as other manifestations [7, 9, 11, 14, 15].

Symptoms arising from these manifestations can impact health‐related quality of life (HRQoL) and lead to poor clinical outcomes [16–18]. As a result of recurrent, often severe, sinopulmonary infections, people with APDS can experience persistent symptoms such as cough, sore throat and bodily pains [14, 17, 19]. Recurrent respiratory tract infections are reported almost universally in APDS and often lead to lung damage, including bronchiectasis [1, 9, 11, 14]. Overall, the high symptom burden, along with the lifestyle adjustments needed to minimise infection risk, continuous medical treatment, and frequent hospital visits, can impact HRQoL for individuals with APDS across physical, social and emotional domains [16, 17, 20, 21].

Several treatments for APDS have been evaluated in clinical trial programs [22, 23], including leniolisib, a PI3Kδ inhibitor which, as of submission, has been approved in the United States (US), the United Kingdom (UK), Israel and Australia [24–27]. Clinical trials used measures of lymphoproliferation (measured via imaging) and B and T cell immunophenotyping to evaluate the treatment effect of leniolisib, with the rationale that improvement of these measures signifies correction of PI3Kδ hyperactivity and is expected to predict clinical benefit [28–30]. However, empirical evidence of this relationship has not yet been formally evaluated; such evidence is important to optimise study designs evaluating experimental and existing therapies. Therefore, we investigated the association of short‐term disease activity endpoints with longer‐term clinical outcomes to support the clinical use of these short‐term disease activity endpoints and to inform potential definitions of treatment response in APDS.

## 2. Results

### 2.1. Baseline Characteristics

In total, 39 patients were included in our investigation (Figure S1). Thirty‐seven patients were included from Study 2201 (NCT02435173), which was a two‐part study investigating leniolisib in patients aged ≥12 years with APDS; it included a phase 2 dose‐finding study (part 1) and a phase 3 randomised controlled trial (RCT: part 2; NCT02435173), each of which was 12 weeks in duration. Of the 37 patients included in Study 2201, 35 continued into its ongoing open‐label extension (OLE), Study 2201E1 (NCT02859727), from which up to 6 years of follow‐up were available. A further two patients were recruited into the OLE to give 37 patients in both studies and 39 overall.

Table 1 presents the baseline characteristics of the cohort; pairwise regression showed no associations between baseline clinical outcomes and baseline disease activity endpoints.

**Table 1:** Baseline characteristics of patients with APDS included in the investigation.

Baseline characteristics	Study 2201 part 1: leniolisib treatment (*N* = 6)	Study 2201 part 2: leniolisib treatment^a^ (*N* = 20)	Study 2201 part 2: placebo treatment^a^ (*N* = 9)	Study 2201 OLE: all patients (*N* = 37)^b^
Male, *n* (%)	4 (66.7)	11 (55.0)	4 (44.4)	21 (56.8)
Age, years, median (IQR)	22 (17, 25)	20 (15, 29)	18 (16, 28)	20 (16, 28)
Race, *n* (%)				
White	6 (100.0)	17 (85.0)	6 (66.7)	31 (83.8)
Black	0	1 (5.0)	1 (11.1)	2 (5.4)
Asian	0	1 (5.0)	1 (11.1)	2 (5.4)
Other	0	1 (5.0)	1 (11.1)	2 (5.4)
Geography, *n* (%)				
US	4 (66.7)	10 (50.0)	6 (66.7)	20 (54.1)
Europe	2 (33.3)	10 (50.0)	3 (33.3)	17 (45.9)
Clinical outcomes, mean (SD)				
PtGA	62.0 (21.9)	55.7 (21.6)	66.6 (20.3)	59.6 (21.2)
SF‐36: PCS	47.4 (8.9)	45.6 (7.2)	47.5 (7.5)	46.4 (7.4)
SF‐36: MCS	48.0 (8.1)	47.9 (8.5)	47.6 (8.6)	47.8 (8.2)
Disease activity endpoints, mean (SD)				
IgM (g/L)	4.1 (2.4)	3.5 (4.5)	3.6 (4.6)	3.7 (4.1)
Naïve B cells (% of total B cells)	31.8 (14.0)	35.2 (16.7)	39.5 (17.4)	35.8 (16.2)
Transitional B cells (% of total B cells)	38.1 (15.1)	26.7 (17.3)	37.0 (18.5)	31.7 (17.6)
Senescent CD8^+^ T cells (% of total CD8^+^ T cells)	26.1 (10.3)	15.8 (8.3)	13.0 (10.8)	17.0 (10.3)
Index lymph node size (cm^2^)	12.5 (8.1)	14.5 (10.5)	18.2 (19.5)	15.1 (12.7)
Spleen volume (cm^3^)	680.1 (231.6)	558.3 (324.0)	483.6 (319.1)	562.3 (306.3)

*Note*: Lower clinical outcome scores and higher disease activity values (except for naïve B cells, where the value is lower) are typically observed in patients with APDS. Baseline defined as per Table S6.

Abbreviations: APDS, activated phosphoinositide 3‐kinase delta syndrome; CD, cluster of differentiation; IgM, immunoglobulin M; IQR, interquartile range; MCS, mental component score; OLE, open‐label extension; PCS, physical component score; PtGA, patient global assessment; SD, standard deviation; SF‐36, 36‐item short form survey; US, United States.

^a^Study 2201 part 2 group contains 20 patients previously randomised to leniolisib (minus one who did not enter the OLE), and nine patients previously randomised to placebo (minus one who did not enter the OLE), as per Figure S1.

^b^This column includes two newly recruited patients who entered directly into OLE, as per Figure S1.

### 2.2. Single Time Point Analysis

#### 2.2.1. Associations Between Change From Baseline in Disease Activity Endpoints and Clinical Outcomes

For the single time point analysis, pairwise associations between changes in disease activity endpoints and clinical outcomes from baseline to 12 weeks (Day 85), irrespective of treatment, were first explored graphically and then modelled using linear regression modelling (Figure S2).

The disease activity endpoints analysed included four measures of immune deficiency: serum IgM levels, naïve B cells (as a percentage of total B cells), transitional B cells (as a percentage of total B cells) and senescent CD8^+^ T cells (as a percentage of total CD8^+^ T cells); and two measures of immune dysregulation: index lymph node size (based on sum of the product of diameters) and three‐dimensional (3D) spleen volume.

Four clinical outcomes were analysed: patient global assessment (PtGA) of disease activity, the 36‐item short form survey (SF‐36) physical component score (PCS) and mental component score (MCS), and infection rate.

An increase in naïve B cells and a decrease in senescent CD8^+^ T cells from baseline to Day 85 were significantly associated with an improvement in PtGA (Table S1); analysis showed patient naïve B cell and senescent CD8^+^ T cell values were below and above the normal range at baseline, respectively (though all patients were included). A decrease in index lymph node size was also significantly associated with an improvement in PtGA. Furthermore, a decrease in spleen volume was significantly associated with an improvement from baseline in the SF‐36: MCS (Table S1). Correlations varied in strength, with a moderate correlation between naïve B cells and PtGA (*r* = 0.49; *p* = 0.006) and a weak correlation between index lymph node size and PtGA (*r* = −0.36, *p* = 0.034).

An additional analysis was conducted comparing nominal values of naïve B cells and PtGA at Day 252 of the OLE, adjusted for baseline PtGA. At Day 252, absolute value of naïve B cells had a strong correlation with PtGA (*r* = 0.70, *p* = 0.027).

No other significant associations were identified in the single time point analysis.

### 2.3. Repeated Measures Analysis

#### 2.3.1. Associations Between Change From Baseline in Disease Activity Endpoints and Clinical Outcomes

In the repeated measures analysis, pairwise associations between the disease activity endpoints and clinical outcomes were analysed across all time points in the combined RCT and OLE dataset, at or up to Day 252 of the OLE. The repeated measures were first explored graphically and then modelled using random effects regression.

The regression coefficients from the repeated measures analysis were generally consistent with the single time point analysis. Multiple disease activity endpoints were significantly associated with an increase in PtGA, including an increase in the percentage of naïve B cells and decreases in IgM, the percentage of transitional B cells, the percentage of senescent CD8^+^ T cells and spleen volume (Table 2).

**Table 2:** Pairwise associations between the clinical outcomes and disease activity endpoints to Day 252 of the OLE for the multiple time point analysis.

Disease activity endpoints	Coefficient	Lower CI	Upper CI	*p*‐Value
Clinical outcome: PtGA				
IgM	−1.65	−3.05	−0.25	**0.021**
Naïve B cells	0.24	0.13	0.35	**<0.001**
Transitional B cells	−0.34	−0.49	−0.19	**<0.001**
Senescent CD8^+^ T cells	−0.77	−1.13	−0.41	**<0.001**
Index lymph node size (cm^2^)	−0.34	−0.70	0.01	0.059
Spleen volume (cm^3^)	−0.03	−0.05	−0.01	**0.043**
Clinical outcome: SF‐36: PCS				
IgM	−0.31	−0.75	0.12	0.158
Naïve B cells	0.03	−0.01	0.07	0.207
Transitional B cells	−0.02	−0.07	0.03	0.438
Senescent CD8^+^ T cells	−0.15	−0.28	−0.02	**0.025**
Index lymph node size (cm^2^)	−0.10	−0.23	0.03	0.137
Spleen volume (cm^3^)	−0.01	−0.02	0.00	0.088
Clinical outcome: SF‐36: MCS				
IgM	0.05	−0.53	0.64	0.859
Naïve B cells	0.04	−0.01	0.10	0.121
Transitional B cells	−0.08	−0.15	−0.01	**0.021**
Senescent CD8^+^ T cells	−0.09	−0.25	0.07	0.247
Index lymph node size (cm^2^)	−0.11	−0.27	0.06	0.203
Spleen volume (cm^3^)	−0.01	−0.02	−0.00	**0.048**

*Note*: Disease activity coefficients are from random effects analyses. An increase of naïve B cells signifies improvement; a decrease of all other disease activity endpoints signifies improvement for all clinical outcomes. Analysis includes patients from RCT part 1, part 2 and the OLE, *N* numbers are shown in Table S5. Baseline was defined as the first baseline prior to study treatment, as shown in Table S6. *p*‐Values in bold are statistically significant.

Abbreviations: CD, cluster of differentiation; CI, 95% confidence interval; IgM, immunoglobulin M; MCS, mental component score; PCS, physical component score; PtGA, patient global assessment; SF‐36, 36‐item short form survey.

A decrease in the percentage of senescent CD8^+^ T cells was significantly associated with an increase in the SF‐36: PCS, while decreases in the percentage of transitional B cells and spleen volume were significantly associated with an increase in the SF‐36: MCS (Table 2). In both PtGA and SF‐36: PCS, significant changes from baseline were observed over the analysis period (Table S2).

#### 2.3.2. Multivariable Analysis

The strength of association of multiple disease activity endpoints with PtGA up to Day 252 of the OLE was investigated. The fixed part of multivariable random‐effects regression modelling for PtGA demonstrated that decreases in the percentage of transitional B cells (coefficient: −0.23; 95% confidence interval [CI]: −0.41 to −0.05, *p* = 0.014) and senescent CD8^+^ T cells (coefficient: −0.77; 95% CI: −1.19 to −0.35, *p* < 0.001) independently predicted a 1‐point increase in the PtGA scale (Table S3).

#### 2.3.3. Infection Rates

The relationship between change in disease activity endpoints from baseline to Day 252 of the OLE and long‐term infection rates in patients treated with leniolisib (>1 year) was explored.

**Figure 1:**
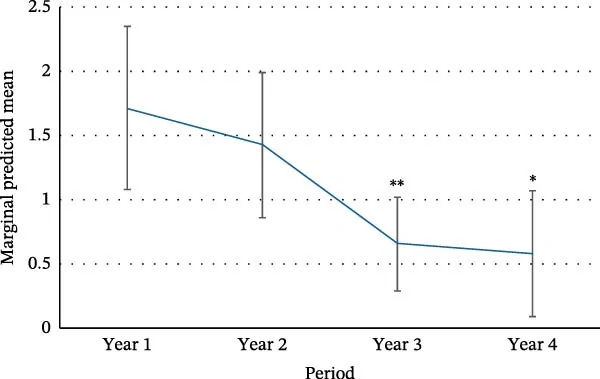
Annualised infection rates in patients treated with leniolisib from the fitted model. Infection rate over the first 365 days from baseline (Year 1) was the reference period. The mean marginal predicted infection rate is shown, with error bars shown as 95% CIs.  ^∗^ *p* < 0.05 compared to year 1 and  ^∗∗^ *p* < 0.01 compared to year 1. Analysis includes patients from RCT part 1, part 2 and the OLE, *N* numbers are shown in Table S5. Baseline was defined as the first baseline prior to study treatment, as shown in Table S6, except for patients who initially received placebo and switched to leniolisib upon entering the OLE, where baseline was redefined as their date of entry into the OLE study. CIs, confidence intervals.

Infection rates decreased from baseline, though there was no significant difference between the infection rates in Year 1 and Year 2 (Figure 1). However, the infection rates dropped significantly in Year 3 and Year 4, relative to Year 1 (by 61.6% and 66.1%, respectively). The infection rates resulting from the model were subsequently Year 1: 1.71 (95% CI: 1.08–2.35), Year 2: 1.43 (95% CI: 0.86–1.99), Year 3: 0.66 (95% CI: 0.29–1.02) and Year 4: 0.58 (95% CI: 0.09–1.07), with an overall *p*‐value of 0.001 (*p*‐value for Year 1: N/A; Year 2: 0.398, Year 3: 0.001 and Year 4: 0.011).

**Figure 2:**
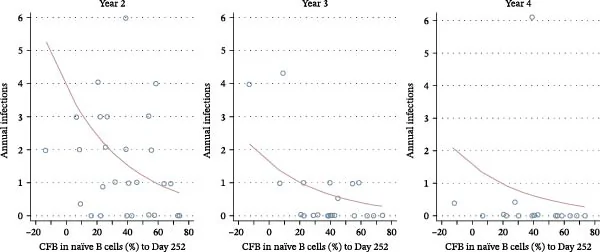
Relationship between change from baseline in naïve B cells to Day 252 of the OLE and year‐specific infection rates (Years 2–4 of follow‐up). Analysis includes patients from RCT part 1, part 2 and the OLE, *N* numbers are shown in Table S5; only patients treated with leniolisib for >1 year were included to control for the confounding effect of the treatment. Baseline was defined as the first baseline prior to study treatment, as shown in Table S6, except for patients who initially received placebo and switched to leniolisib upon entering the OLE, where baseline was redefined as their date of entry into the OLE study. CFB, change from baseline; OLE, open‐label extension.

Change in proportion of naïve B cells to Day 252 was significantly associated with annualised infection rates; our analysis of the relationship indicated that each 1‐percentage point increase in the proportion of naïve B cells was associated with a corresponding 2.3% reduction in yearly infection rates (Table 3 and Figure 2). All other disease activity endpoints showed no association with annualised infection rates. In the multivariable analysis, all disease activity endpoints, except naïve B cells, were removed from the final model using backward stepwise regression, as they did not reach the significance threshold (0.05); data from this analysis are presented in Table S4.

**Table 3:** Multilevel model estimates of the effect of disease activity endpoint changes from baseline to Day 252 of the OLE on annualised infection rates.

Disease activity endpoint	*N*	IRR	Lower CI	Upper CI	*p*‐Value
IgM	32	1.008	0.919	1.107	0.860
Naïve B cells	28	0.977	0.966	0.988	**<0.001**
Transitional B cells	24	1.009	0.987	1.032	0.415
Senescent CD8^+^ T cells	32	0.998	0.962	1.037	0.934
Index lymph node size (cm^2^)	30	1.007	0.977	1.038	0.643
Spleen volume (cm^3^)	31	1.000	0.997	1.003	0.955

*Note*: The overall annualised infection rates predicted by these endpoint changes are presented across the 4 years of follow‐up in Figure 1, and were year 1: 1.71 (95% CI: 1.08–2.35), year 2: 1.43 (95% CI: 0.86–1.99), year 3: 0.66 (95% CI: 0.29–1.02) and year 4: 0.58 (95% CI: 0.09–1.07). Baseline was defined as the first baseline prior to study treatment, as shown in Table S6, except for patients who initially received placebo and switched to leniolisib upon entering the OLE, where baseline was redefined as their date of entry into the OLE study. Analysis includes patients from RCT part 1, part 2 and the OLE. *N* refers to the specific number of patients with a measurement of each disease activity endpoint at Day 252. *p*‐Values in bold indicate statistically significant longitudinal treatment effects on change from baseline in disease activity endpoints.

Abbreviations: CD, cluster of differentiation; CI, 95% confidence interval; IgM, immunoglobulin M; IRR, incidence rate ratio; OLE, open‐label extension.

## 3. Discussion

Short‐term measures of disease activity are needed to evaluate treatment effect and response and to accelerate access to innovative therapies in rare and life‐threatening diseases [31, 32]. While normalisation of some aspects of immune system function may occur rapidly in APDS upon initiating leniolisib treatment, restoration of immune competence with an accompanying reduction in the risk of severe symptoms can take years to observe [15, 29, 33]. This is the first study to present consistent associations between short‐term disease activity endpoints and longer‐term clinical outcomes and HRQoL in patients with APDS. The use of clinical trial data to explore and quantify these associations using both longitudinal and cross‐sectional approaches generates evidence that demonstrates an association between disease activity endpoints and clinical outcomes in APDS [34].

Immune deficiency and dysregulation in APDS cause multi‐systemic manifestations, leading to high symptom burden and significantly impacted HRQoL [16, 17, 20]. Previous publications from the leniolisib RCT have reported on improvements in lymphoproliferative and immunological outcomes following leniolisib treatment, with significant changes in the co‐primary endpoints (reduction in diameter of index lymph nodes and increase in naïve B cells out of total B cells) at Day 85 of the RCT, alongside a significant reduction in splenomegaly and improvements in key immune cell subsets, which were maintained in the OLE [28, 29]. The original RCT publication presents reference ranges for these outcomes, demonstrating that patients initially had values below these ranges [29]. The data in this manuscript indicate that the improvements in lymphoproliferative outcomes (measured via imaging) and immunological outcomes (measured by B and T cell immunophenotyping) with leniolisib translate into improvements in clinical outcomes, consistent with views expressed by clinicians and regulatory bodies [24, 30] and supporting the favourability of these immunological and lymphoproliferative outcomes. For immunological outcomes, improvement in patient assessment of HRQoL was significantly associated with changes in naïve B cells, IgM levels, transitional B cells and senescent CD8^+^ T cells. Lymphoproliferative outcomes were associated with improvements in HRQoL, with decreases in lymph node size and spleen volume showing statistically significant correlation with improvements in PtGA and SF‐36: MCS, respectively, in single time point analysis, and spleen volume associated with PtGA and SF‐36 in the repeated measures analysis. In addition to presenting with organ dysfunction where lymphoproliferation occurs, patients with lymphoproliferative conditions, such as APDS and other IEIs, may be at a higher risk of developing lymphoma than those without [35], due to an interplay between lymphoproliferation, reduced immune system function and an increase in B‐cell proliferation [36]. The potential for reductions in lymphoproliferation to have a longer‐term impact on malignancy risk, alongside the reductions in senescent and exhausted T cells observed in the pivotal study of leniolisib [29], requires further study.

Although recurrent and chronic infections are a hallmark of APDS and affect most patients, with chronic infections reported in the majority of patients at baseline in the clinical trials and similar findings observed across natural history studies [29, 37, 38], the annualised infection rates observed in this analysis were numerically lower than what might be expected. Infection rates declined from an average of 1.43 in Year 2 to 0.66 in Year 3, before remaining stable to Year 4. While year‐to‐year variation may partly reflect chance, as would be expected amongst a healthy population, the overall trend of decline is consistent with other studies of leniolisib [39–41]. While pre‐treatment infection rate data were not collected in Study 2201, a separate publication including six patients from the study reported a mean pre‐leniolisib infection rate of approximately four per year, falling to a mean of 3 in year one and 0.2 in year six of follow‐up [40]. A recent retrospective analysis of US pharmacy data found a significant intra‐patient reduction in infection rates when comparing pre‐ and post‐leniolisib treatment periods [41]. Additionally, comparison of leniolisib‐treated patients with controls from the European Society for Immunodeficiencies (ESID)‐APDS registry found a significant reduction in annualised infection rates in the leniolisib arm versus those receiving standard of care [39], further supporting the ability of leniolisib to reduce infections. In our investigation, the observed reduction in long‐term infection rates was significantly associated with an increase and stabilisation of naïve B cells to Day 252 of the OLE, consistent with clinician and regulatory views of this endpoint in APDS [24, 30, 42]. Survivor bias is not thought to contribute to the observed reduction in long‐term infection rates as there was only one non‐treatment‐related death and minimal discontinuations [28]. Conversely, time‐on‐treatment effects may have contributed to the observed reduction; further time‐on‐treatment‐adjusted analyses could explore this further. Alternatively, the observed continued reduction in infection rates may be explained by the fact that naïve B cells, as well as other immune cell subsets, have been shown in other studies to return to normal ranges within 12 weeks of treatment and then plateau, contributing to immune system reconstitution; this, in turn, explains the continued reduction in infection rates during longer‐term treatment without additional increases in naïve B cell levels [28]. Similar effects have been observed after haematopoietic stem cell transplant, when infection rates continue to decrease for several years after the procedure due to the time taken for complete immune reconstitution, which is expected to take 2–5 years [43, 44]. Hence, more heterogeneity in infection rates is seen in earlier years before full reconstitution occurs, and therefore, further reductions in infection rates can be expected over longer time frames. Reduction in long‐term infection rates, together with the biological rationale for the association of disease activity endpoints with long‐term clinical outcomes, supports the use of these endpoints in clinical trials, observational studies and as part of response criteria to inform treatment decisions.

Our analysis was guided by principles for associating short‐ and long‐term outcomes published by the European Network for Health Technology Assessment (EUnetHTA) and Institute for Quality and Efficiency in Health Care (IQWiG), which were designed for evaluating conditions with relatively large prevalence (e.g., diabetes and breast cancer) [34, 45]. However, when conducting clinical trials in rare and ultra‐rare diseases, the ability to detect treatment effects on long‐term clinical outcomes and validate significant relationships remains a challenge due to small participant pools, short trial durations, impractical trial designs, incomplete understanding of disease natural history and the absence of best practices [46]. For example, no association was identified between change in transitional B cells and the SF‐36: MCS in the single time point analysis, while a significant negative association was observed in the repeated measures analysis; this demonstrates that significant associations can arise when more data are included in the analyses. Therefore, a non‐statistically significant relationship between a disease activity endpoint and a clinical outcome is not definitive evidence of the absence of a relationship. This means that rare and ultra‐rare diseases often need to rely on identifying a correlation between endpoints at the individual patient level, as presented in this paper, and/or biological plausibility evidence [46]. While the analyses presented provide supportive associative evidence, they do not validate the endpoints described as surrogate markers; as with many rare and ultra‐rare diseases, validation in line with established guidance may not be possible.

Further study with more data may further strengthen relationships found in this study, as well as reveal relationships that have not yet been characterised. This highlights the need for high quality real‐world data to be used in combination with clinical trial data to improve understanding of patient experiences in APDS and responses to treatment, particularly given the impracticality of conducting long‐term RCTs in rare and ultra‐rare diseases. In addition, APDS is a multi‐systemic disease with heterogenous clinical presentation that may impact HRQoL in different ways; for example, patients with irreversible lung damage may have a different HRQoL response to treatment than patients with resolving gastrointestinal symptoms [14, 17].

The results of this study are broadly consistent with the limited clinical data in other studies of APDS. A small, 12‐week, phase 1b study of seletalisib (a PI3Kδ inhibitor) for APDS (*N* = 7) observed an increase in naïve B cells along with reductions in patient‐reported disease activity and improvement in some clinical features, such as reduction of lymphadenopathy, improvement in lung function, enteropathy and thrombocytopenia [22]. This suggests that short‐term clinical improvements associated with other PI3Kδ inhibitors may correlate with long‐term outcomes in APDS. Collectively, these results highlight that, when clinical data are limited, disease activity endpoints can provide valuable insights into the potential longer‐term improvements in clinical outcomes for individuals with APDS who are treated with PI3Kδ inhibitors.

Considering IEIs beyond APDS, similar correlations between short‐ and long‐term clinical outcomes have been observed; for example, studies of common variable immunodeficiency (CVID), a heterogeneous immune defect which can present with similar manifestations to APDS [14, 47], have found significant associations between naïve B cells and clinical features. One study of 47 patients with CVID (2008–2012) found that CD8^+^ T cell and memory B cell counts were significantly lower in people with an interstitial lung disease (ILD) score >5, which was clinically significant [48]. Another study of 49 children with CVID (1995–2011) compared groups with accumulated transitional B cells (and lower proportion of mature B cells including naïve B cells) with people with accumulated naïve B cells (with lower proportion of more‐mature B cells), and found that the group with lower naïve B cells had significantly higher prevalence of enteropathy (55.6% vs 12.5%, *p* = 0.0201), granuloma formation (33.3% vs 0%, *p* = 0.0154, with most cases in the lungs), production of monoclonal or oligoclonal IgM (44.4% vs 8.3%, *p* = 0.0342), as well as combined features of cytopenia and lymphoproliferation or cytopenia and enteropathy (both 44.4% vs 4.2%, *p* = 0.0133) [49]. It is worth noting that CVID populations may include a considerable proportion of monogenic IEIs, potentially including APDS and other related disorders, particularly as CVID is a common APDS misdiagnosis [2], and as the cohorts in these analyses were presented prior to APDS classification in 2013, APDS patients may be included. Nevertheless, these data provide evidence that the correlation between short‐ and long‐term clinical outcomes has been seen in other IEIs, and therefore, disease activity endpoints may be suitable measures of treatment response in these broader conditions.

Finally, the use of disease activity endpoints could extend beyond informing potential definitions of treatment response in APDS and broader IEIs. For example, using measures of immune deficiency and dysregulation as disease activity endpoints in APDS could provide insight into the natural history of untreated individuals by quantifying baseline activity and longitudinal change in disease, characterising disease progression.

## 4. Data Limitations and Perspectives

Key limitations of our analyses reflect those which are common when studying rare and ultra‐rare diseases, including the small number of patients, which limits the power and generalisability to all individuals living with APDS. There is a risk that this study was underpowered to detect significant associations, especially in cross‐sectional analysis. Additionally, as multiple tests were performed across several endpoints and multiplicity was not adjusted for in these pairwise comparison analyses, results should be interpreted with caution as it is plausible that some statistically significant findings are due to chance.

## 5. Conclusions

This is the first study exploring and quantifying the relationships between disease activity endpoints, including serum IgM levels, naïve B cells, transitional B cells, senescent CD8^+^ T cells, index lymph node size and spleen volume, and conventional longer‐term measures of HRQoL in patients with APDS. The results indicate that short‐term improvements in these markers were associated with long‐term improvements in infection rates and patient well‐being in our clinical trial setting, and should be considered within development programmes for APDS and for evaluating response to treatment in individuals with APDS.

## 6. Materials and Methods

### 6.1. Study Design and Patients

This analysis used data from the clinical development programme of leniolisib in adolescents and adults (aged 12 years and older) with APDS; this consisted of Study 2201 (NCT02435173), a two‐part study including a phase 2 dose‐finding study (part 1) and phase 3 RCT (part 2), each 12 weeks in duration, and Study 2201E1, an ongoing OLE (NCT02859727) with up to 6 years of follow‐up at the data cut‐off. Full details of the design and results of these studies have been published previously [28, 29, 50]. The dataset used for the analysis had a cut‐off date of 13^th^ March 2023.

#### 6.1.1. Baseline Definitions

Two baseline definitions were utilised depending on the analysis. The standard baseline was defined as the ‘first baseline’ prior to any study treatment, which was dependent on the endpoint: for PtGA, Physician Global Assessment (PGA), SF‐36, IgM and B and T cell immunophenotyping, this was day 0; for the sum of the product of diameters (SPD) of index lymph nodes and spleen 3D volume, this was a median of 8 days prior to the start (Table S6).

A modified baseline (specifically for annualised infection rate analyses) for patients who initially received placebo in part 2 was defined as their date of entry into the OLE study. This aimed to accurately evaluate the long‐term effects of leniolisib and isolate it from the confounding effects of placebo. Patients who received leniolisib from the beginning of part 1 or part 2 retained their standard baseline (as described in the earlier paragraph).

### 6.2. Outcomes

#### 6.2.1. Disease Activity Endpoints

Four measures of immune deficiency, which are expected to change rapidly and which are hallmarks of APDS, were included as disease activity endpoints: serum IgM levels, naïve B cells (defined by CD27^−^ and CD10^−^; expressed as a percentage of total B cells), transitional B cells (defined by CD27^−^ and CD10^+^; as a percentage of total B cells) and senescent CD8^+^ T cells (defined by CD57^+^ and CD8^+^; as a percentage of total CD8^+^ T cells) [1, 7, 9, 11, 14, 51]. Two measures of immune dysregulation were also included as disease activity endpoints: index lymph node size (based on the SPD) and spleen volume (3D; based on computed tomography [CT]/magnetic resonance imaging [MRI] images). In most people with APDS, all of these measures of disease activity are elevated, except for naïve B cells, which are reduced.

Immune deficiency endpoints were collected at baseline and regularly during part 1, part 2, and the OLE study, at or up to Day 252 of the OLE [52, 53]. Immune dysregulation endpoints were collected at baseline and at Day 84 of part 1, Day 85 of part 2, and Day 168 or 252 of the OLE [52, 53]. *N* numbers for each endpoint are shown in Table S5, with timepoints shown in Table S6.

#### 6.2.2. Clinical Outcomes

Four clinical outcomes were included: PtGA of disease activity, the SF‐36 PCS and MCS, and infection rate. All clinical outcomes were regularly collected as outcomes during part 1, part 2, and the OLE study; specifically, Day 84 of part 1, Day 85 of part 2, and Day 168 or 252 of the OLE. *N* numbers for each outcome are shown in Table S5, with timepoints shown in Table S6.

The PtGA is an assessment that uses a visual analogue scale (VAS) to assess disease from the patient perspective [54]. In these studies, patients were asked to mark a point on a 100 mm VAS anchored between ‘very poor’ (corresponding to 0 points) and ‘very good’ (100 points) in relation to the question: ‘considering all the ways APDS/PASLI (p110δ‐activating mutation causing senescent T cells, lymphadenopathy and immunodeficiency) affects you, please indicate with a vertical mark through the horizontal line how well you are doing’.

The SF‐36 measures eight scales scoring physical functioning, role‐physical, bodily pain, general health, vitality, social functioning, role‐emotional and mental health. The first four scales are combined to derive the PCS and the latter four to derive the MCS [55]. Higher scores indicate a better health state.

Infections were also measured by including any adverse event with the MedDRA system organ class ‘infections and infestations’, with a start date recorded between baseline and the final study day. Infection numbers were calculated for each year of follow‐up during the study. As placebo treatment was only administered for 12 weeks, differential treatment effects were not analysed and follow‐up under placebo was excluded from analysis of infection rates.

### 6.3. Statistical Analysis

Pairwise associations between the six disease activity endpoints and four clinical outcomes (PtGA, SF‐36: PCS, SF‐36: MCS, and infection rate) were investigated. Both single time point and repeated measures analyses were conducted.

#### 6.3.1. Single Time Point Analysis

The single time point analysis evaluated associations between changes in disease activity endpoints and clinical outcomes, irrespective of treatment, between baseline and 12 weeks using linear regression modelling. An additional analysis was conducted comparing nominal values of naïve B cells and PtGA at Day 252 of the OLE, adjusted for baseline PtGA.

#### 6.3.2. Repeated Measures Analysis

The longitudinal associations between disease activity endpoints and clinical outcomes were analysed across all time points in the combined RCT and OLE dataset, at or up to Day 252 of the OLE, using a random effects multilevel regression model, accounting for time‐varying treatment administration. Models were fitted for each clinical outcome‐disease activity endpoint pair to investigate if there was a statistically significant association between the clinical outcome and disease activity endpoint.

#### 6.3.3. Multivariable Analysis

The strength of association of multiple endpoints with long‐term PtGA was investigated using a multivariable repeated measures random‐effects regression model. The model was fitted using the backward stepwise approach. The initial models included all disease activity endpoint variables. At each step, the variable with the highest *p*‐value was removed and the estimates were reassessed until only variables with *p*‐value <0.05 remained in the model. The final models with significant predictors of PtGA are presented.

#### 6.3.4. Infection Rates

The relationship between change in disease activity endpoint values from baseline to Day 252 of the OLE and long‐term infection rates (through up to 4 years of treatment with leniolisib) in patients treated with leniolisib for >1 year was explored by using a mixed‐effects negative binomial model. Only patients treated with leniolisib for >1 year were included to control for the confounding effect of the treatment. The strength of association of multiple endpoints with infection rates was assessed using backward stepwise mixed‐effects negative binomial regression. The baseline visits in patients treated with placebo during the part 2 study were set at entry into the OLE study, thus excluding the time of treatment with placebo.

#### 6.3.5. Significance Levels and Statistical Software

Missing and incomplete data are reported in the results tables. Data were analysed using the complete case approach. All statistical inference used a significance level of 0.05 and a corresponding two‐sided 95% CI as a measure of uncertainty. The statistical software STATA/BE 17.0 for Windows (StataCorp LLC) and R version 4.3.0 were used for the analysis.

**Nomenclature**
3D: Three‐dimensional
APDS: Activated phosphoinositide 3‐kinase delta syndrome
CD: Cluster of differentiation
CI: Confidence interval
CMV: Cytomegalovirus
CT: Computed tomography
CVID: Common variable immunodeficiency
EBV: Epstein–Barr virus
HRQoL: Health‐related quality of life
IEI: Inborn error of immunity
IgM: Immunoglobulin M
ILD: Interstitial lung disease
IQWiG: Institute for Quality and Efficiency in Health Care
MCS: Mental Component Score
MRI: Magnetic resonance imaging
OLE: Open‐label extension
PASLI: P110δ‐activating mutation causing senescent T cells, lymphadenopathy and immunodeficiency
PCS: Physical Component Score
PGA: Physician Global Assessment
PI3Kδ: Phosphoinositide 3‐kinase delta
PtGA: Patient Global Assessment
RCT: Randomised controlled trial
SD: Standard deviation
SF‐36: 36‐Item Short Form Survey
SPD: Sum of the product of diameters
UK: United Kingdom
US: United States
VAS: Visual analogue scale.

## Author Contributions

Joanne Tutein Nolthenius, Louise Linsell, Neil Hawkins, Yuxian Chen, Barbara Torlinska, John Whalen and Anna Šedivá made substantial contributions to the conception and design of the study, the analysis and interpretation of the data, the drafting of the article or its critical revision for important intellectual content, and the final approval of the version of the article published.

## Funding

This work was supported by the Pharming Group N.V.

## Disclosure

The authors have no disclosures to declare. Author contributions are outlined above, and conflicts of interests are below.

## Ethics Statement

Original studies/registries obtained appropriate ethics approval.

## Consent

Patient consent was obtained for participation in the study and publication of the article.

## Conflicts of Interest

Joanne Tutein Nolthenius is an employee of Pharming. Louise Linsell, Neil Hawkins and Yuxian Chen are employees of Visible Analytics, who received funding from Pharming to carry out the data analysis in this study. Barbara Torlinska was an employee of Visible Analytics at the time of study conduct, who received funding from Pharming to carry out the data analysis in this study. John Whalen is an employee and stock holder of Pharming. Anna Šedivá is a consultant and lecturer to Pharming, Takeda and Octapharma, and is also lecturer to CSL Behring.

## Supporting Information

Additional supporting information can be found online in the Supporting Information section.

## Supporting information


**Supporting Information** The supporting information for this manuscript contain the following figures and tables. Figure S1: Study flow diagram from the two‐part phase 2 trial and phase 3 RCT, with the OLE. Figure S2. Relationship between change from baseline to Day 85 in clinical outcomes and change from baseline to Day 85 in disease activity endpoints. Table S1: Disease activity endpoints modelling the univariable relationships with clinical outcome: single time point linear regression. Table S2: Repeated measures analysis of clinical outcomes and disease activity endpoints change from baseline to Day 252 of the OLE. Table S3: Fixed part of multivariable random‐effects regression modelling for PtGA. Table S4: Annualised infection rate model: the effect of change from baseline to Day 252 of the OLE in the percentage of naïve B cells. Table S5: Number of patients with outcome reports in the clinical trial of leniolisib. Table S6: Time points used in the longitudinal analysis of PRO‐surrogate outcomes.

## Data Availability

The following will be made available upon request: study protocol, statistical analysis plan, text, tables, figures and appendices. Researchers who have a methodologically sound research proposal should send inquiries or requests to info@pharming.com. Data requestors may be required to sign a data access agreement.
