# Supplementary material for: Associations Between Short‐Term Changes in Disease Activity and Long‐Term Clinical Outcomes in Activated Phosphoinositide 3‐Kinase Delta Syndrome
Source: J Immunol Res. 2026 Sep 16;2026:1486611. doi: 10.1155/jimr/1486611 (PMC13583054; doi:10.1155/jimr/1486611)
Supplement: Supplementary file 1 [file JIMR-2026-1486611-s001.docx]

SUPPORTING INFORMATION

Figure S1. Study flow diagram from the two-part Phase 2 trial and Phase 3 RCT, with the OLE

 **Footnotes:** Two patients did not proceed to the OLE from Part 2 due to personal choice or death.
**Abbreviations:** OLE: open-label extension; n: number of participants; RCT: randomised controlled trial.

**Figure S2. Relationship between change from baseline to Day 85 in clinical outcomes and change from baseline to Day 85 in disease activity endpoints**

**Footnotes:** A negative correlation between elevated disease activity endpoints (IgM, transitional B cells, senescent T cells, index lymph node size, and spleen volume) and clinical outcomes, along with a positive correlation between the reduced disease activity endpoint (naïve B cells) and clinical outcomes, suggests an improved disease state. Analysis includes patients from RCT Part 1 and Part 2, N numbers are shown in **Table S5**. Baseline was defined as the first baseline prior to study treatment. Two patients with substantial hyper-IgM at baseline saw large reductions in IgM over time; supplementary analyses to remove these outliers (not shown) demonstrated a negative correlation was seen with or without outliers included. **Abbreviations:** IgM: immunoglobulin M; MCS: Mental Component Score; PCS: Physical Component Score; PtGA: Patient Global Assessment; SF-36: 36-Item Short Form Survey.

**Table S1. Disease activity endpoints modelling the univariable relationships with clinical outcome: single time point (Day 85) linear regression**

| **Disease activity endpoints** | **Coefficient** | **Lower CI** | **Upper CI** | **p-value** |
| --- | --- | --- | --- | --- |
| **Clinical outcome – PtGA** | | | | |
| IgM^a^ | –1.93 | –4.41 | 0.54 | 0.122 |
| Naïve B cells | 0.55 | 0.17 | 0.94 | **0.006** |
| Transitional B cells | –0.49 | –0.98 | 0.01 | 0.055 |
| Senescent CD8^+^ T cells | –1.59 | –2.62 | –0.55 | **0.004** |
| Index lymph node size (cm^2^) | –1.17 | –2.25 | –0.09 | **0.034** |
| Spleen volume (cm^3^) | –0.03 | –0.08 | 0.02 | 0.184 |
| **Clinical outcome – SF-36: PCS** | | | | |
| IgM | –0.13 | –0.86 | 0.60 | 0.719 |
| Naïve B cells | 0.04 | –0.08 | 0.16 | 0.460 |
| Transitional B cells | 0.01 | –0.14 | 0.16 | 0.932 |
| Senescent CD8^+^ T cells | –0.12 | –0.45 | 0.22 | 0.483 |
| Index lymph node size (cm^2^) | –0.22 | –0.51 | 0.08 | 0.146 |
| Spleen volume (cm^3^) | –0.01 | –0.02 | 0.01 | 0.326 |
| **Clinical outcome – SF-36: MCS** | | | | |
| IgM | –0.33 | –1.41 | 0.75 | 0.544 |
| Naïve B cells | 0.07 | –0.11 | 0.25 | 0.440 |
| Transitional B cells | –0.02 | –0.25 | 0.21 | 0.861 |
| Senescent CD8^+^ T cells | –0.38 | –0.88 | 0.11 | 0.124 |
| Index lymph node size (cm^2^) | –0.39 | –0.84 | 0.06 | 0.090 |
| Spleen volume (cm^3^) | –0.03 | –0.04 | –0.01 | **0.012** |

**Footnotes:** P-values in bold are statistically significant. An increase in naïve B cells and a decrease in all other disease activity endpoints signifies improvement in clinical outcomes. ^a^One patient’s IgM observation was identified as an outlier, which was 4.9 standard deviations (SDs) away from the mean of IgM change from baseline to Day 85. When excluding this observation, the linear regression results modelling clinical outcomes with IgM showed a decrease in IgM levels was significantly associated with an improvement in PtGA from baseline to Day 85 (coefficient: –5.39 [95% confidence interval (CI): –9.76 to –1.02], p=0.017). Analysis includes patients from RCT Part 1 and Part 2, N numbers are shown in **Table S5**. Baseline was defined as the first baseline prior to study treatment.
**Abbreviations:** CD: cluster of differentiation; CI: 95% confidence interval; IgM: immunoglobulin M; MCS: Mental Component Score; PCS: Physical Component Score; PtGA: Patient Global Assessment; SF-36: 36-Item Short Form Survey.

Table S2. Repeated measures analysis of clinical outcomes and disease activity endpoints change from baseline to Day 252 of the OLE

|  | **Coeff.** | **Lower CI** | **Upper CI** | **p-value** |
| --- | --- | --- | --- | --- |
| **Clinical outcomes:** | | | | |
| PtGA | 9.70 | 4.35 | 15.04 | **<0.001** |
| SF-36: PCS | 3.10 | 1.55 | 4.65 | **<0.001** |
| SF-36: MCS | 1.97 | –0.17 | 4.11 | 0.071 |
| **Disease activity endpoints:** | | | | |
| IgM | –1.34 | –2.17 | –0.51 | **0.002** |
| Naïve B cells | 25.66 | 21.32 | 29.99 | **<0.001** |
| Transitional B cells | –16.86 | –21.50 | –12.23 | **<0.001** |
| Senescent CD8^+^ T cells | –5.20 | –7.15 | –3.25 | **<0.001** |
| Index lymph node size (cm^2^) | –6.21 | –8.78 | –3.64 | **<0.001** |
| Spleen volume (cm^3^) | –168.56 | –208.63 | –128.49 | **<0.001** |

**Footnotes:** P-values in bold are statistically significant. An increase signifies improvement for naïve B cells; a decrease signifies improvement for all other disease activity endpoints. Analysis includes patients from RCT Part 1, Part 2 and the OLE, N numbers are shown in **Table S5**. Baseline was defined as the first baseline prior to study treatment, shown in **Table S6**. **Abbreviations:** CD: cluster of differentiation; CI: 95% confidence interval; IgM: immunoglobulin M; MCS: Mental Component Score; PCS: Physical Component Score; PtGA: Patient Global Assessment; SF-36: 36-Item Short Form Survey.

Table S3. Fixed part of multivariable random-effects regression modelling for PtGA to Day 252 of the OLE

|  | **PtGA** | | | |
| --- | --- | --- | --- | --- |
|  | **Coeff.** | **Lower CI** | **Upper CI** | **p-value** |
| **Transitional B cells** | –0.23 | –0.41 | –0.05 | **0.014** |
| **Senescent CD8^+^ T cells** | –0.77 | –1.19 | –0.35 | **<0.001** |
| Intercept | 1.81 | –4.12 | 7.74 | 0.549 |

**Footnote:** P-values in bold are statistically significant. A decrease in transitional B cell and senescent CD8^+^ T cells signifies improvement for PtGA. Analysis includes patients from RCT Part 1, Part 2 and the OLE, N numbers are shown in **Table S5**. Baseline was defined as the first baseline prior to study treatment, shown in **Table S6**. **Abbreviations:** CD: cluster of differentiation; CI: 95% confidence interval; Coeff.: coefficient; IgM: immunoglobulin M; PtGA: Patient Global Assessment.

Table S4. Annualised infection rate model: the effect of change from baseline to Day 252 of the OLE in the percentage of naïve B cells

|  | **Treated** | | | |
| --- | --- | --- | --- | --- |
|  | **IRR** | **Lower CI** | **Upper CI** | **p-value** |
| **Naïve B cells** | **0.977** | **0.966** | **0.988** | **0.001** |
| Year 1 | 1.000 |  |  |  |
| Year 2 | 0.816 | 0.509 | 1.307 | 0.398 |
| Year 3 | 0.337 | 0.178 | 0.636 | 0.001 |
| Year 4 | 0.325 | 0.136 | 0.774 | 0.011 |
| Intercept | 1.754 | 1.072 | 2.872 | 0.025 |

**Footnotes:** P-values in bold are statistically significant. Analysis includes patients from RCT Part 1, Part 2 and the OLE, N numbers are shown in **Table S5**. Baseline was defined as the first baseline prior to study treatment, shown in **Table S6**, except for patients who initially received placebo and switched to leniolisib upon entering the OLE, where baseline was redefined as their date of entry into the OLE study. **Abbreviations:** CI: 95% confidence interval; IRR: incidence rate ratio; OLE: open-label extension.

Table S5. Number of patients with outcome reports in the clinical trial of leniolisib

|  | **IgM** | **Naïve B cells** | **Transitional B cells** | **Senescent T cells** | **Spleen 3D volume** | **SPD of index lymph nodes** | **PtGA** | **PGA** | **SF-36** |
| --- | --- | --- | --- | --- | --- | --- | --- | --- | --- |
| **RCT Part 1 (N=6)** | | | | | | | | | |
| Baseline | 6 | 6 | 6 | 6 | 6 | 6 | 6 | 6 | 6 |
| Day 1 | 6 | 6 | 6 | 6 | - | - | - | - | - |
| Day 29 | 6 | 6 | 6 | 5 | - | - | 6 | 6 | 6 |
| Day 57 | 6 | 6 | 6 | 6 | - | - | 6 | 6 | 6 |
| Day 84 | 5 | 5 | 5 | 6 | 6 | 6 | 6 | 6 | 6 |
| **RCT Part 2 (N=31)** | | | | | | | | | |
| Baseline | 31 | 29 | 27 | 26 | 30 | 31 | 31 | 31 | 31 |
| Day 1 | 31 | 29 | 28 | 25 | - | - | - | - | - |
| Day 29 | 30 | 28 | 28 | 26 | - | - | 31 | 31 | 31 |
| Day 57 | 31 | 28 | 29 | 24 | - | - | 31 | 31 | 31 |
| Day 85 | 31 | 26 | 26 | 25 | 29 | 31 | 31 | 31 | 31 |
| **OLE (N=37)** | | | | | | | | | |
| Day 1^a^ | 33 | 33 | 34 | 31 | - | - | 37 | 31 | 31 |
| Day 84^a^ | 37 | 33 | 31 | 30 | - | - | 37 | 32 | 32 |
| Day 168^a^ | 36 | 33 | 32 | 32 | 20 | 18 | 37 | 31 | 32 |
| Day 252^a^ | 37 | 29 | 27 | 24 | 12 | 12 | 36 | 32 | 31 |

**Footnotes:** Patients with missing data due to not receiving a measurement at that time point; ^a^Days calculated from start of OLE study. **Abbreviations:** IgM: immunoglobulin M; OLE: open-label extension; PGA: Physician Global Assessment; PtGA: Patient Global Assessment; RCT: randomised controlled trial; SF-36: 36-Item Short Form Survey; SPD: sum of product of diameters.

Table S6. Time points used in the longitudinal analysis of PRO-surrogate outcomes

| **Trial** | | **Median time from first baseline (IQR)** | |
| --- | --- | --- | --- |
| **RCT Part 1** | **RCT Part 2** | **PtGA, PGA, SF-36, IgM, B and T cells^a^** | **SPD of index lymph nodes, spleen 3D volume^b^** |
| Baseline | Baseline | 0 days (0 to 0) | -8 days (-16 to 0) |
| Day 29 | Day 29 | 29 days (28 to 29) | - |
| Day 57 | Day 57 | 57 days (56 to 57) | - |
| Day 84 | Day 85 | 85 days (84 to 85) | 86 days (85 to 86) |
| **OLE** | |  | |
| Day 84^c^ | | 168 days (167 to 196) = 5.5 months | - |
| Day 168^c^ | | 253 days (248 to 282) = 8.3 months | - |
| Day 252^c^ | | 339 days (335 to 356) = 11.1 months | 283 days (252 to 340) = 9.3 months |

**Footnotes:** ^a^Timing reported based on the PtGA, which may have differed slightly for a small number of other tests; ^b^SPD and Spleen 3D were mostly measured based on the same schedule. However, there were some minimal differences, values are reported based on the SPD; ^c^Days calculated from start of OLE study. **Abbreviations:** IgM: immunoglobulin M; IQR: interquartile range; OLE: open-label extension; PGA: Physician Global Assessment; PtGA: Patient Global Assessment; RCT: randomised controlled trial; SF-36: 36-Item Short Form Survey; SPD: sum of product of diameters.
